# From Annotation to Prediction: Hospital-Grade Early Seizure Risk Prediction from Adult EEG

**DOI:** 10.3390/diagnostics16030492

**Published:** 2026-02-05

**Authors:** Norah Alharbi, Mashael Aldayel, Shrooq Alsenan, Raneem Alyami, Enas Almowalad, Eman Alkethiry

**Affiliations:** 1 Department of Internal Medicine, College of Medicine, Princess Nourah bint Abdulrahman University, P.O. Box 84428, Riyadh 11671, Saudi Arabia; noaalharbi@pnu.edu.sa; 2 Information Technology Department, College of Computer and Information Sciences, King Saud University, Riyadh 11543, Saudi Arabia; maldayel@ksu.edu.sa; 3 Information Systems Department, College of Computer and Information Sciences, Princess Nourah bint Abdulrahman University, P.O. Box 84428, Riyadh 11671, Saudi Arabia; 444008722@pnu.edu.sa; 4 Neurosciences Section, King Abdullah Bin Abdulaziz University Hospital, Princess Nourah bint Abdulrahman University, Riyadh 11671, Saudi Arabia; esalmowalad@kaauh.edu.sa (E.A.); eralkethiry@kaauh.edu.sa (E.A.)

**Keywords:** epilepsy, electroencephalography (EEG), epilepsy monitoring unit (EMU), artificial intelligence (AI), seizure prediction

## Abstract

**Background**: Manual review of EEG recordings in clinical settings is inherently time-consuming and labor-intensive. These challenges highlight a pressing need for automated EEG analysis tools capable of supporting clinicians by improving efficiency and diagnostic accuracy. **Objectives**: This study aims to develop and validate an AI-based model for the automated interpretation of adult EEG recordings. Unlike previous approaches that emphasize seizure detection during ictal states, our model targets the early prediction of seizure risk through systematic annotation and recognition of interictal patterns. **Methods**: The model is designed to accurately distinguish between normal and abnormal EEGs, encompassing both interictal and ictal activity. Abnormal EEGs will be further classified into three clinically relevant categories: (1) non-epileptiform abnormalities such as focal or diffuse slowing, (2) epileptiform discharges, and (3) electrographic seizures. Three AI-based classification algorithms were implemented: Support Vector Machine (SVM), Random Forest (RF), and K-Nearest Neighbors (KNN). **Results**: RF demonstrated optimal performance across most tasks, achieving 96.50% accuracy for normal activity identification. This AI-driven system enhances the efficiency, consistency, and accessibility of EEG interpretation. It is particularly valuable in settings with limited access to neurophysiologists and offers an innovative approach to improving diagnostic timelines and clinical decision-making. **Conclusions**: Ultimately, this tool will support physicians in diagnosing neurological conditions and monitoring patient progress over time.

## 1. Introduction

Epilepsy is one of the most prevalent and serious neurological disorders, affecting over 70 million individuals worldwide, with an estimated prevalence of 7.6 per 1000 persons [1,2,3]. It is characterized by recurrent, brief, and unpredictable disruptions in behavior or perception, caused by excessive synchronization of neuronal activity within cortical networks [4]. These epileptic seizures vary widely in their clinical presentation and duration from one individual to another [4,5]. Seizure onset can originate from different brain regions, and the associated clinical manifestations often depend on the underlying location and physiological role of the affected neuronal circuits.

Multiple neurophysiological and imaging modalities have been developed for epilepsy diagnosis, including electroencephalography (EEG), magnetoencephalography (MEG), and functional magnetic resonance imaging (fMRI) [6]. Among these, EEG remains the gold standard due to its ability to record real-time electrical activity of the brain [7]. EEG plays a central role in distinguishing epileptic seizures from non-epileptic paroxysmal events and transient alterations in consciousness. Different recording protocols are used in clinical practice, ranging from routine 20–30 min EEGs to prolonged monitoring, long-term video EEG in epilepsy monitoring units (EMUs), and continuous EEG (cEEG) in intensive care settings [1,6].

Recent evidence underscores the expanding clinical utility of EEG and cvEEG beyond traditional seizure detection, supporting their integration into the evaluation of diverse neurological and systemic disorders [8]. As these modalities become more widely adopted in emergency and inpatient settings, the volume of EEG recordings requiring timely expert review has increased substantially, creating a growing mismatch between clinical demand and the availability of specialized interpreters [9]. In many healthcare systems, EEGs are frequently read by general neurologists rather than fellowship-trained neurophysiologists, which may further contribute to interpretive variability and diagnostic delays.

The challenge is compounded by the intrinsic complexity of EEG interpretation. Normal physiological patterns and benign variants can mimic pathological activity, and artifacts—such as muscle activity, eye movements, or sharply contoured waveforms—may appear deceptively similar to epileptiform discharges. These factors increase the risk of inaccurate classification of EEG findings, potentially leading to overdiagnosis, underdiagnosis, or misdiagnosis of epilepsy [10,11,12]. The problem is particularly pronounced in resource-limited settings or institutions without access to trained neurophysiologists, where interpretive errors may carry significant clinical consequences.

Even in hospitals with dedicated neurophysiology services, the interpretation of EEG remains a significant operational challenge. Manual review of EEG recordings, whether in critically ill patients undergoing continuous monitoring in EMUs or intensive care units (ICUs), or in routine outpatient settings, is inherently time-consuming and labor-intensive. The increasing volume of EEG studies further amplifies this burden, often resulting in delayed reporting and prolonged clinical decision-making.

The integration of artificial intelligence (AI) into EEG interpretation offers a powerful solution to the growing clinical and operational challenges associated with manual review. Advanced machine learning and deep learning algorithms can rapidly and systematically detect ictal and interictal abnormalities, including subtle or subclinical patterns that may escape human recognition. In high-acuity settings such as intensive care units, AI-assisted EEG can provide real-time alerts and flag suspicious segments to support the timely detection of electrographic seizures and avert clinical deterioration. Additionally, in outpatient care, automated detection of interictal epileptiform discharges and focal slowing may accelerate epilepsy diagnosis, inform earlier treatment decisions, and provide objective markers for monitoring response. Overall, AI-enhanced EEG analysis can reduce interpretive burden while improving the consistency of clinically relevant event detection for specialist review.

This study presents a multi-class AI framework for the automated interpretation of adult EEG recordings, with a specific focus on classifying abnormal EEG activity into clinically meaningful categories. The proposed system not only detects seizure activity but also identifies a broader spectrum of pathological features, including interictal abnormalities such as focal or diffuse slowing and epileptiform discharges, offering a more comprehensive diagnostic tool than those focused solely on ictal events. By enabling early recognition of abnormal EEG activity, this approach supports timely clinical intervention, potentially mitigating severe seizure-related outcomes and enhancing patient management in high-acuity environments, including neurology wards, epilepsy monitoring units, and intensive care units.

Furthermore, in this study, we exclusively utilized real-world clinical EEG recordings acquired from routine patient care, including data from intensive care unit settings, rather than relying on public or synthetic datasets. All EEG recordings were systematically annotated across background activity, interictal features, and ictal events when present, resulting in a richly detailed dataset grounded in real clinical practice. To the best of our knowledge, this work represents one of the first regionally based initiatives to develop an AI-driven EEG classification model trained on hospital-derived adult EEG data, highlighting the importance of locally curated clinical datasets in advancing AI-assisted neurophysiology.

## 2. Literature Review

The current literature review surveys recent advances in EEG-based seizure detection, with a focus on contemporary feature extraction techniques and the evolution of machine learning and deep learning models that aim to enhance the automated recognition of epileptic activity. This review outlines the strengths, limitations, and clinical implications of prior work.

### 2.1. Clinical Prediction Models and Risk Stratification in Epilepsy

Recent work in epilepsy has increasingly focused on prediction models that use demographic, clinical, imaging, and electrophysiological features to estimate seizure risk and treatment outcomes. At a population level, Luo et al. conducted a systematic review and meta-analysis of epilepsy prediction models for children and adolescents, reporting that most existing models are limited by small sample sizes, high risk of bias, and inadequate external validation, and that few models incorporate EEG features in a structured way [13].

Complementing this, Ratcliffe et al. systematically reviewed clinical prediction models for treatment outcomes in newly diagnosed epilepsy, using PROBAST to show that most models suffer from methodological weaknesses, including poor handling of missing data and overfitting, and often lack transparent reporting [14]. Both reviews highlight that while predictive modeling is increasingly common, robust, generalizable models that translate into routine clinical practice remain scarce, particularly models that leverage rich EEG phenotypes.

Within hospital and ICU practice, a central advance has been the development of EEG-based risk scores to estimate short-term seizure probability. Struck et al. introduced the 2HELPS2B score, a parsimonious model combining five EEG patterns with a clinical seizure history to stratify cEEG patients into graded risk levels, with seizure probabilities ranging from 5% to >95% [15]. The score was later validated in a large multicenter cohort, confirming its calibration and discriminative performance and providing practical recommendations on EEG monitoring duration for different risk categories [16].

Building on this work, Hsiao et al. recently demonstrated that 2HELPS2B can be applied to routine EEG (rEEG) in the medical ICU, showing that higher scores are strongly associated with post-rEEG seizure occurrence and that a cutoff ≥ 2 optimally predicts subsequent seizures and informs antiseizure medication decisions [17]. These studies show the feasibility and clinical impact of standardized EEG-based seizure risk scores, but they primarily rely on a fixed set of human-interpreted features and focus on near-term electrographic seizure occurrence, rather than richer multi-class labeling of interictal patterns for early risk prediction.

### 2.2. Automated EEG Interpretation and Seizure Detection

Parallel to clinical scoring systems, a large body of work has focused on automated EEG analysis using signal processing and machine learning to detect epileptic seizures and classify normal vs. abnormal EEG. Early work concentrated on hand-crafted features—time-domain statistics, frequency-domain features (e.g., power spectral density), and time–frequency representations such as wavelets or short-time Fourier transforms—combined with classical machine learning classifiers (e.g., SVMs, RFs). A recent comprehensive review by El-Shoka et al. summarized these approaches, highlighting common feature extraction pipelines, evaluation datasets (e.g., Bonn, CHB-MIT), and persistent limitations such as overreliance on small datasets, data leakage, and lack of external validation [7].

To move closer to hospital-grade practice, Tveit et al. developed the SCORE-AI system, training convolutional neural networks on large cohorts of routine clinical EEG to classify recordings as normal or abnormal and to further classify abnormal recordings into subtypes (e.g., epileptiform focal, epileptiform generalized, nonepileptiform focal, nonepileptiform diffuse), demonstrating performance comparable to expert readers and illustrating the potential for large-scale automated EEG reporting in real-world settings [18].

Several contemporary studies using the Bonn dataset illustrate the typical feature-engineering paradigm. Usman et al. combined discrete wavelet transform, entropy measures, and statistical descriptors with ensemble learning classifiers to improve binary seizure detection performance [19]. Liu et al. explored hybrid time–frequency features and dimensionality reduction to enhance classification robustness [20].

Kunekar et al. proposed a multi-channel approach using wavelet packet decomposition and statistical features, reporting improved accuracy in distinguishing seizure from non-seizure EEG [21]. These studies underscore the importance of careful feature extraction and selection but are generally limited to binary classification (seizure vs. non-seizure) and rely on small, often single-center datasets.

More recently, deep learning has enabled end-to-end feature learning from raw or minimally processed EEG. Khan et al. transformed EEG segments into time–frequency images using the continuous wavelet transform and applied convolutional neural networks to predict focal seizures, showing that CNNs can effectively exploit rich time–frequency structure for preictal detection [22]. Oliva et al. leveraged multitaper spectral features fed into deep neural networks for binary and multiclass EEG classification, demonstrating that robust spectral estimates can improve downstream classification performance [23]. Mallick et al. proposed a 1D CNN combined with bidirectional LSTM and GRU units to simultaneously capture local morphological details and longer-range temporal dependencies in EEG, achieving high accuracy on the Bonn dataset [24]. Zaid et al. introduced a preprocessed and combined EEG dataset that merges multiple public datasets and applied deep CNNs for seizure classification, showing that data harmonization and augmentation can substantially improve generalization [25]. Berrich and Guennoun proposed a hybrid CNN–SVM and DNN–SVM framework with PCA-based feature reduction, reporting strong performance on EEG-based epilepsy detection [26].

Collectively, these works demonstrate that both classical and deep learning models can achieve high accuracy in seizure detection and EEG abnormality classification, but several limitations persist: (i) many studies use small, highly curated datasets not representative of real-world hospital EEG; (ii) tasks are often simplified to binary detection; and (iii) interictal patterns such as epileptiform discharges and focal slowing, which are critical for early risk assessment, are frequently collapsed into undifferentiated “abnormal” classes, limiting their direct clinical interpretability.

## 3. Materials and Methods

**Figure 1:**
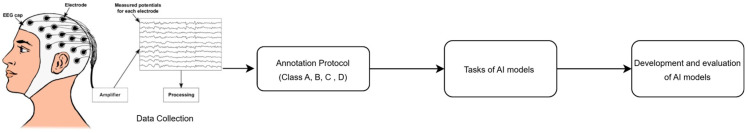
Research framework, where (Class A = Normal, Class B = Slowing, Class C = epileptiform discharges., Class D = elec-trographic seizures (generalized or focal).

We designed a framework for AI-based seizure detection tailored for adult EEG recordings. The proposed framework includes data collection, the annotation protocol, AI models’ implementation, and evaluation, as shown in Figure 1.

### 3.1. Dataset Collection

This study was conducted at King Abdullah bin Abdulaziz University Hospital (KAAUH) in Riyadh, Saudi Arabia. We collected EEG recordings acquired over a 10-year period (2014–2024) from a range of clinical settings, including routine outpatient EEG evaluations, continuous video-EEG monitoring (cvEEG), and ICU-based EEG surveillance. All EEGs were recorded using the Cadwell Arc system (250 Hz sampling rate), employing a standard 10–20 electrode configuration with the following 19 scalp electrodes: Fp2, F4, C4, P4, O2, F8, T4, T6, Fz, Cz, Pz, Fp1, F3, C3, P3, O1, F7, T3, and T5, supplemented by A1/A2 references.

A total of 28 subjects were enrolled in the dataset. Each EEG recording was independently reviewed by three board-certified neurophysiologists, with a total of six expert reviewers participating in the annotation process to improve inter-rater reliability and ensure diagnostic accuracy. The final dataset used for training and validating the AI-based classification model comprised expert-labeled EEG segments with high clinical fidelity.

### 3.2. Annotation Protocol

To standardize and streamline the annotation of EEG recordings for model training, we developed a hierarchical classification system organized into four major diagnostic categories: normal EEG, abnormal slowing, epileptiform discharges, and seizures. Each category was further subdivided based on clinical presentation and anatomical localization, and all entries were assigned both literal codes to support human-readable analysis and machine learning integration, as shown in Table 1.

Class A (Normal) included recordings interpreted as normal background activity. Class B (Slowing) captured generalized slowing and focal slowing. Class C represented epileptiform discharges. Class D included electrographic seizures (generalized or focal) arising from specific brain hemispheres. This detailed annotation scheme ensured that the dataset was structured in a way that reflects both clinical relevance and regional specificity, providing an ideal foundation for training interpretable AI models for automated EEG interpretation.

### 3.3. Tasks of AI Models

The primary objective of training the AI model was to develop a robust classifier capable of distinguishing between normal and abnormal adult EEG recordings, and further stratifying the latter into clinically meaningful subcategories. The classification schema was based on predefined EEG classes (A–D), as follows:

1. Task 1: Identification of normal brain activity (Class A).
2. Task 2: Detection of abnormal brain activity (Classes B–D).
3. Task 3: Differentiation between normal activity (Class A) and interictal abnormalities (separated Classes B–D).
4. Task 4: Differentiation between normal activity (Class A) and ictal events (Class D).
5. Task 5: Differentiation between interictal (combined Classes B and C) and ictal activity (Class D).

The task design was adapted from research [27]. These tasks were designed to systematically assess the model’s performance, beginning with the identification of normal brain activity and progressively advancing to the more complex differentiation of various abnormal states, including interictal and ictal events. The development and evaluation of AI models is described in the following section.

### 3.4. Development and Evaluation of AI Models

**Figure 2:**
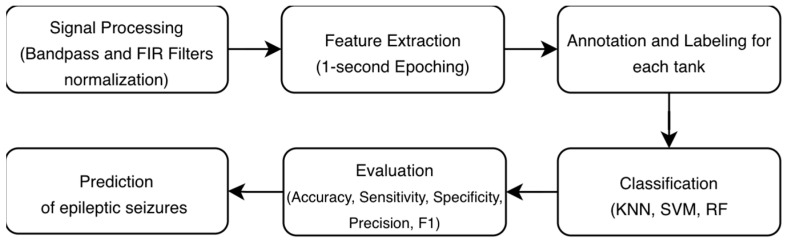
Methodology for automated EEG-based seizure detection.

This section outlines in detail the development and evaluation of AI models in EEG signals. The proposed methodology illustrates the complete processing pipeline for automated EEG-based seizure detection. The process is structured around four main stages: signal processing, feature extraction, annotation/labeling based on the task, classification, and evaluation, as shown in Figure 2.

1. Signal Preprocessing

Continuous EEG data were preprocessed using MNE-Python 3.12. The raw data were band-pass-filtered between 0.5 Hz and 40 Hz to attenuate low-frequency drift and high-frequency muscle or environmental noise. A zero-phase finite impulse response (FIR) filter was designed using the Hamming window method (firwin) to prevent temporal distortion of the signals. The filter had a length of 1651 samples (6.604 s) with transition bandwidths of 0.50 Hz and 10.00 Hz at the lower and upper cut-offs, respectively.

Additionally, this research employed a data preprocessing pipeline where categorical labels were first encoded numerically using LabelEncoder to facilitate AI and machine learning algorithms. We then normalized the EEG features using MinMaxScaler normalization to transform the 23-channel EEG data with 250 timepoints per trial into a [0,1] range. This scaling reduced amplitude disparities across electrodes and improved training stability.

2. Feature Extraction

This study employed a comprehensive feature extraction pipeline utilizing epoching and discrete wavelet transform (DWT) with db4 wavelet to decompose EEG signals into five canonical frequency bands: delta (0.5–3 Hz), theta (4–7 Hz), alpha (8–13 Hz), beta (14–30 Hz), and gamma (31–45 Hz). Continuous EEG data were segmented into 1 s fixed-length epochs and analyzed at the epoch level to capture short-duration EEG patterns in continuous monitoring settings. This approach is commonly adopted in EEG-based seizure detection and classification studies [24,25]. Epochs containing annotations were retained for analysis, while unannotated epochs were discarded. For each annotated epoch, the annotation description was extracted and used as the class label. Epochs from all recording files were concatenated into a single data structure for subsequent analysis. The data structure shape was (17,403, 23, 250), which represents 17,403 epochs/trials, 23 channels, and 250 timepoints per trial.

For each of the 23 EEG channels within every epoch, we extracted 16 distinct statistical features from the wavelet coefficients of each frequency band, encompassing basic statistics (mean, standard deviation, variance, median, mean absolute value, and maximum absolute value), energy metrics (total energy and average power), higher-order moments (skewness and kurtosis), information-theoretic measures (Shannon entropy), temporal characteristics (zero-crossing rate and waveform length), and Hjorth parameters (activity, mobility, and complexity). To create epoch-level feature vectors suitable for machine learning classification, we computed the mean across all 23 channels for each feature-band combination, resulting in a compact yet information-rich 80-dimensional feature vector per epoch (5 bands × 16 features). This aggregation approach preserves the spectral characteristics captured by wavelet decomposition while reducing dimensionality and mitigating potential overfitting, ensuring computational efficiency for subsequent classification tasks. The resulting feature matrix of dimensions (17,403 epochs × 80 features) provides a robust foundation for discriminating between experimental conditions while maintaining interpretability through direct correspondence to neurophysiologically meaningful frequency bands and signal properties.

3. Annotation and Labeling

To create a labeled dataset, we employed an annotation-driven epoching strategy. Rather than using all available data, we specifically extracted 1-s epochs that contained experimental annotations, as these markers indicated periods of neurophysiological relevance, which are four main classes: A, B, C, and D. Table 1 provides a description of each class. Since EEG signals were segmented into epochs with a duration of approximately 1 s, the complete dataset consisted of nearly 17,403 epochs. The distribution of these class labels was imbalanced, with Class A being the most frequent (*n* = 5957), followed by Class B (*n* = 3313), Class D (*n* = 2996), and Class C (*n* = 372).

**Figure 3:**
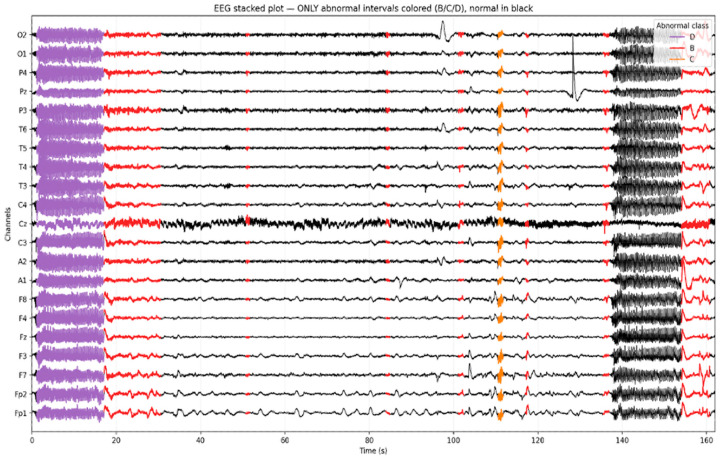
Example of a multi-channel EEG segment (≈500 s) with model-predicted intervals.

To visually validate the annotation of the temporal EEG, we generated a stacked EEG plot highlighting the intervals classified as abnormal. As shown in Figure 3, normal EEG activity (Class A) is rendered in black, while distinct color overlays represent the model-predicted abnormalities. Seizure segments (Class D) are marked in purple, epileptiform discharges (Class C) in orange, and slowing (Class B) in red. This visualization illustrates the ability to localize and differentiate pathological EEG patterns over time and across channels. Importantly, it confirms that focal abnormalities such as spikes or rhythmic slowing are not only correctly identified by the model but also temporally aligned with visually recognizable events on the EEG traces.

Figure 3 presents a 500+ second segment of a multi-channel EEG recording with intervals labeled by the AI model. Normal EEG segments are displayed in black, while color-coded overlays indicate detected abnormalities: purple for Class D (seizure activity), orange for Class C (epileptiform discharge), and red for Class B (slowing).

4. Classification models and Evaluation

We developed multiple classification models, namely Support Vector Machine (SVM), Random Forest (RF), and K-Nearest Neighbors (KNN), for EEG-based seizure detection. These models were selected due to their proven effectiveness in discriminating seizure from non-seizure EEG activity and their widespread use as benchmark classifiers in automated EEG analysis [28,29,30].

SVM performs by non-linearly mapping input vectors into a high-dimensional feature space where an optimal hyperplane is constructed to separate classes with the maximal margin. This balances model complexity against training error to minimize the generalization error on unseen data. The decision surface is determined solely by a small subset of training data called support vectors, while slack variables are introduced to handle non-separable data by penalizing misclassifications. This formulation allows the SVM to create complex, non-linear decision boundaries efficiently using kernel functions, which compute dot products in a high-dimensional space without requiring explicit mapping [31]. SVM was implemented using its default hyperparameter configuration (regularization parameter C = 1.0 and the kernel coefficient gamma = ‘scale’). The radial basis function kernel was used, which is a non-linear kernel suitable for capturing complex, non-linear decision boundaries. This choice allows the model to learn decision boundaries directly from the scaled EEG features while avoiding unnecessary complexity, ensuring a fair comparison with other classifiers.

RF is an ensemble learning method that constructs a large collection of decision trees, where each tree depends on the values of a random vector sampled independently and with the same distribution for all trees. The algorithm combines bagging (Bootstrap Aggregating)—where each tree is trained on a random sample of the data with replacement—with random feature selection, where the best split at each node is chosen from a randomly selected subset of features rather than all features. By incorporating randomness at both the sampling and feature selection levels, the algorithm reduces the correlation between individual trees without compromising their accuracy. This ensures that as the forest grows, the generalization error stabilizes at a specific value rather than increasing, effectively making the model immune to overfitting [32]. Based on this principle, the Random Forest classifier was implemented with 500 decision trees to improve model stability and reduce variance. Given that EEG features are often high-dimensional and noisy, increasing the number of trees leads to more consistent predictions while limiting overfitting, resulting in stable performance across different EEG classes [33].

KNN is a nonparametric discrimination procedure that classifies an unknown data point based on the majority category of its *k* closest observations in a training set. This method effectively performs local density estimation without requiring prior knowledge of the data’s underlying statistical distributions [34]. The KNN algorithm was configured with the number of neighbors set to *k* = 1 to preserve fine-grained local patterns in the EEG feature space. Since EEG segments can exhibit subtle differences between normal and pathological activity, using a single nearest neighbor emphasizes the closest matching pattern without smoothing important signal variations [35].

A subject-wise splitting strategy was implemented across all classification tasks to prevent data leakage. Unlike random sample-based partitioning, this approach ensures complete separation between training and testing subjects, with test subjects comprising approximately 20% of the total subject pool (4–5 subjects per task). This methodology mimics the clinical reality where models must generalize to entirely new patients, providing a more stringent evaluation of model robustness. The resulting test sets ranged from 1366 to 3222 EEG samples while preserving within-subject class distributions. This splitting strategy represents a critical methodological strength, as it prevents artificial performance inflation that can occur when samples from the same subject appear in both training and testing partitions.

Moreover, we used five key metrics—accuracy, sensitivity, specificity, precision, and F1 score—to comprehensively assess the performance of classification models in EEG-based seizure detection. Accuracy provided an overall measure of classification correctness, while sensitivity specifically quantified the model’s ability to correctly identify true seizure events, which is critically important in medical applications where missing actual seizures could have serious consequences. Specificity measured the effectiveness in recognizing non-seizure periods, ensuring minimal false alarms, and precision evaluated the reliability of positive seizure predictions by calculating the proportion of true seizures among all predicted seizures. Finally, the F1 score offered a balanced harmonic mean between precision and sensitivity, providing a single metric that is particularly valuable for evaluating performance on imbalanced datasets where seizure events are typically rare compared to normal EEG activity.

We acknowledge that class imbalance is an inherent challenge in clinical EEG datasets. In this study, we reported the weighted F1 score as a primary metric, which calculates the average F1 score weighted by the number of true instances for each class. This provides a more representative performance measure than accuracy in the presence of imbalance. Moreover, Random Forest models inherently mitigate imbalance through their ensemble and weighting mechanisms.

## 4. Results and Discussion

The results were generated based on a hierarchical set of five classification tasks (defined in Section 3.3).

The result of the first task is shown in Table 2. The RF model achieved the best overall performance, yielding an accuracy of 96.50% with a good balance between sensitivity (96.50%) and specificity (95.98%). The SVM classifier followed closely with an accuracy of 95.70%, demonstrating high specificity (92.67%) and precision (96.10%), suggesting robust performance in identifying true negatives and minimizing false positives. While KNN showed the lowest performance among the three models, it still achieved a respectable accuracy of 93.24%. These results establish RF as the most robust model for binary EEG classification in subject-independent validation scenarios in clinical settings.

The second classification task aimed to detect abnormal EEG activity corresponding to Classes B (slowing), C (epileptiform discharges), and D (seizures), while explicitly excluding Class A (normal brain function). The dataset used for this analysis thus focused solely on abnormal EEG segments, enhancing the model’s ability to distinguish between various pathological patterns.

As shown in Table 3, RF achieved the highest performance with 86.46% accuracy. The SVM achieved moderate performance (82.72% accuracy), while KNN showed the lowest performance at 71.74% accuracy. These results highlight the increased difficulty of multi-class abnormal EEG classification, particularly for rare event detection. Moreover, the findings reinforce the robustness of the RF classifier, particularly in multi-class EEG abnormality detection tasks.

The third classification task aimed to build an AI system capable of distinguishing between normal EEG activity (Class A), interictal abnormalities comprising slowing and epileptiform discharges (Classes B and C), and ictal activity (Class D), corresponding to active seizure events. This multi-class scenario presents a more complex challenge, reflecting the real-world diagnostic needs of clinical EEG interpretation.

Table 4 presents the results of the third-class classification task encompassing all EEG states (normal, slowing, epileptiform discharges, and seizures). Among the evaluated models, SVM achieved the highest overall accuracy (89.96%) and F1 score (89.44%), indicating superior global classification performance. The RF model demonstrated comparable accuracy (88.66%) and yielded the highest specificity (90.31%), reflecting strong performance in correctly identifying non-pathological EEG segments. KNN classifier showed slightly lower overall accuracy (87.13%) but maintained high precision (88.63%) and F1 score (87.63%).

Despite these favorable aggregate metrics, all models exhibited critically low sensitivity, ranging from 48.75% (RF) to 52.75% (KNN), indicating substantial difficulty in correctly identifying abnormal EEG states. Notably, both SVM and RF failed to detect epileptiform discharges, a key interictal marker essential for epilepsy diagnosis and clinical decision-making. Although KNN demonstrated limited ability to identify this pattern, its performance remained insufficient for reliable clinical application. These results suggest that model performance was driven largely by the correct classification of common EEG patterns, with inadequate detection of rare but clinically critical abnormalities.

The result of the fourth Task is shown in Table 5. This task focused on a binary classification task to distinguish between normal EEG activity (Class A) and ictal events (Class D), representing clinically significant seizure episodes. To isolate this comparison, all interictal abnormalities were excluded from the dataset, specifically Class B and Class C samples. This allowed for a direct evaluation of each model’s ability to identify active seizure patterns against a normal background.

KNN achieved the highest overall performance with 87.43% accuracy and the best-balanced profile across metrics (sensitivity: 83.00%, specificity: 78.57%, F1 score: 86.98%). RF followed with 85.23% accuracy. SVM showed similar limitations, with 83.05% accuracy.

This indicates that while SVM and RF optimize for overall accuracy by prioritizing majority class detection, KNN maintains a better balance between detecting normal and abnormal patterns. The results suggest that for this seizure detection task with moderate class imbalance, simpler distance-based classification may offer more clinically useful performance than complex ensemble or margin-based methods.

The goal of the fifth task was to distinguish between interictal EEG abnormalities, including both slowing and epileptiform discharges (Classes B and C) and ictal activity (Class D). This task is of high clinical relevance, as the ability to accurately differentiate between interictal and ictal events is essential for epilepsy diagnosis and management. To isolate this classification problem, we excluded Class A (*n* = 5957 samples), which corresponds to normal brain function.

Table 6 shows that the RF model outperformed the other classifiers, achieving an accuracy of 85.65% and a good balance between sensitivity (84.00%) and specificity (81.82%). The SVM achieved an accuracy of 77.75%, while KNN performed moderately, with 74.89% accuracy. These results confirm a well-balanced RF model capable of distinguishing ictal from interictal activity with high fidelity.

**Figure 4:**
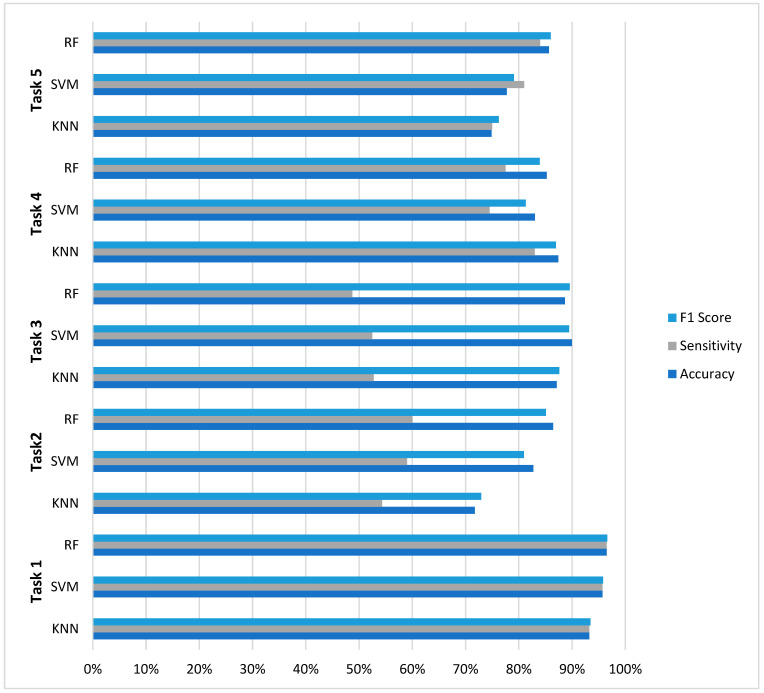
Comparative analysis across five EEG classification tasks.

The evaluation across five distinct EEG classification tasks reveals nuanced performance characteristics that inform model selection for neurophysiological signal analysis. As summarized in Figure 4, RF demonstrated superior overall consistency, achieving optimal performance in three of five classification challenges (Tasks 1, 2, and 5) with accuracy values of 96.50%, 86.46%, and 85.65%, respectively. However, this performance supremacy was not universal: SVM achieved the highest accuracy (89.96%) in Task 3 (four-class classification), while KNN surprisingly outperformed both ensemble and margin-based methods in Task 4 (87.43% accuracy).

A critical finding emerging from this comparative analysis is the pronounced impact of class imbalance on model performance. All models exhibited significant sensitivity degradation in multiclass scenarios with severe class imbalance, where sensitivity values dropped to 48–60% despite accuracy above 86%. The results further reveal distinct model characteristics: RF demonstrated balanced metric profiles with minimal sensitivity-specificity disparity in binary tasks (≤4.5% gap in Tasks 1, 4, and 5), SVM exhibited precision-oriented optimization (achieving the highest precision in 4/5 tasks), while KNN showed unexpectedly competitive performance in moderate imbalance scenarios. These findings collectively suggest that optimal model selection for EEG classification depends on specific task characteristics—particularly class distribution and clinical requirements—rather than the universal superiority of any single algorithm.

These results further confirm the robustness of RF classifiers for clinically nuanced EEG classification tasks and highlight their potential role in assisting neurologists in distinguishing interictal abnormalities from ongoing seizure activity during real-time monitoring. While classical machine learning approaches have yielded encouraging results, this work represents only the initial phase of a broader research effort. Current development is focused on deep learning architectures, including convolutional and recurrent neural networks, designed to model the complex spatiotemporal characteristics of EEG signals and improve performance across heterogeneous patient data.

A primary limitation of this study is the cohort size of 28 subjects. Although our subject-wise splitting strategy prevents data leakage and provides a rigorous internal evaluation, the findings are derived from a single-center dataset. Consequently, the model’s performance may be influenced by subject-specific physiological and pathophysiological signatures present in this cohort, which may limit its direct generalizability to broader, more diverse patient populations. External validation on independent, multi-center datasets is therefore essential to confirm the robustness and clinical applicability of the proposed framework before any potential deployment.

In the upcoming stages, we plan to evaluate the diagnostic validity of the AI system by comparing its predictions with ground truth clinical assessments provided by expert neurophysiologists. In addition to these evaluations, we are preparing to test the system on new, unseen EEG datasets to ensure its robustness across diverse patient populations and recording conditions. This comparative analysis will serve as a critical benchmark for assessing clinical utility. Additional future directions include exploring real-time deployment, assessing model uncertainty, and integrating EEG interpretation with other clinical and imaging data sources. These steps are essential to advancing the system from a research prototype to a practical tool for epilepsy monitoring and broader neurodiagnostic applications.

## 5. Conclusions

This study demonstrates the feasibility and clinical relevance of machine learning-based automated interpretation of adult EEG recordings. Through a comprehensive comparative evaluation, several key insights were identified. Firstly, the Random Forest (RF) classifier emerged as the most robust general-purpose model, showing stable performance across classification tasks of varying complexity, with a mean accuracy of 88.50%. Secondly, class imbalance was found to exert a greater impact on model performance than the number of classes itself; while classifiers maintained high accuracy in balanced multiclass settings, performance deteriorated markedly under imbalanced conditions, irrespective of class count. Thirdly, context-dependent advantages of simpler models were observed, as exemplified by the superior performance of KNN in Task 4 (accuracy 87.43%, sensitivity 83.00%), challenging the assumption that more complex models consistently outperform simpler approaches in EEG classification.

Beyond conventional seizure detection, the proposed AI-based framework enables comprehensive characterization of EEG abnormalities, incorporating both ictal and interictal patterns, including non-epileptiform slowing and epileptiform discharges. The use of systematic, clinically informed annotation supported the development of a multiclass classification paradigm capable of distinguishing normal EEG activity, interictal abnormalities, and ictal events. This approach captures the broad spectrum of EEG pathology encountered in adult clinical practice, particularly in high-acuity settings where subtle or subclinical abnormalities are frequently overlooked. Collectively, these findings underscore the potential of AI-driven EEG analysis to enhance diagnostic accuracy, support timely clinical decision-making, and improve patient management in both routine and critical care environments.

## Figures and Tables

**Table 1:** Description of dataset classes (annotation).

Class	Remarks	Final Interpretation
A	Normal, healthy person	Normal EEG
B	Patient with abnormal EEG Between the seizure episodes (slowing of the brain wave)	Abnormal EEG. Interictal abnormality present
C	Patient with abnormal EEG Showed activity between the seizure episodes (epileptiform discharge)	Abnormal EEG. Interictal abnormality present
D	Patient with abnormal EEG During seizure	Abnormal EEG. Ictal (seizure) activity present

**Table 2:** Identification of normal brain activity (Class A).

Model	Accuracy	Sensitivity	Specificity	Precision	F1 Score
KNN	93.24%	93.24%	85.35%	93.83%	93.45%
SVM	95.70%	95.70%	92.67%	96.10%	95.82%
RF	96.50%	96.50%	95.98%	96.91%	96.61%

**Table 3:** Detection of abnormal brain activity (Classes B, C, and D).

Model	Accuracy	Sensitivity	Specificity	Precision	F1 Score
KNN	71.74%	54.33%	76.83%	76.94%	72.94%
SVM	82.72%	59.00%	79.98%	80.43%	80.97%
RF	86.46%	60.00%	79.66%	84.00%	85.11%

**Table 4:** Differentiation between normal activity (Class A), interictal (separated Classes B and C), and ictal activity (Class D).

Model	Accuracy	Sensitivity	Specificity	Precision	F1 Score
KNN	87.13%	52.75%	80.59%	88.63%	87.63%
SVM	89.96%	52.50%	80.31%	89.14%	89.44%
RF	88.66%	48.75%	90.31%	91.16%	89.57%

**Table 5:** Differentiation between normal activity (Class A) and ictal events (Class D).

Model	Accuracy	Sensitivity	Specificity	Precision	F1 Score
KNN	87.43%	83.00%	78.57%	87.62%	86.98%
SVM	83.05%	74.50%	66.40%	85.54%	81.29%
RF	85.23%	77.50%	70.50%	87.37%	83.94%

**Table 6:** Differentiation between interictal (combined Classes B and C) and ictal activity (Class D).

Model	Accuracy	Sensitivity	Specificity	Precision	F1 Score
KNN	74.89%	75.00%	75.11%	79.96%	76.26%
SVM	77.75%	81.00%	83.62%	84.08%	79.09%
RF	85.65%	84.00%	81.82%	86.69%	86.00%

## Data Availability

The datasets generated and analyzed during the current study are not publicly available due to the presence of identifiable patient information and institutional privacy regulations. De-identified EEG data may be made available from the corresponding author upon reasonable request. Access to the data will require completion of a data use agreement and, when applicable, approval from the relevant institutional review board.
